# *“Google It”:* Experiences of Children and Parents During the Assessment and Diagnosis of Complex Regional Pain Syndrome Type I

**DOI:** 10.1002/pne2.70045

**Published:** 2026-09-05

**Authors:** Emma Evans, Lisa Roberts, Rhiannon Joslin

**Affiliations:** ^1^ School of Health Sciences University of Southampton Southampton UK; ^2^ Therapy Services Department University Hospital Southampton NHS Foundation Trust Southampton UK; ^3^ Women's and Children's Department University Hospitals Sussex, St. Richard's Hospital Chichester UK

**Keywords:** child, chronic pain, complex regional pain syndrome, diagnosis, parent, qualitative

## Abstract

Complex Regional Pain Syndrome Type I (CRPS‐I) is a chronic pain disorder that can affect children most commonly in early adolescence. In comparison to adult literature, there is a sparsity of evidence regarding the effect of CRPS‐I on children. This evidence gap contributes to diagnostic delays, affecting children and parents physically, psychologically, and socially. The experiences of children and their parents when receiving a CRPS‐I diagnosis have yet to be explored. The current study explored the experiences of children and parents during the assessment and diagnosis of CRPS‐I. This qualitative study involved secondary analysis of verbatim interviews with children and their parents from a primary study that explored outcomes of chronic musculoskeletal pain treatment in children aged 11–18 years. Timeline drawings were used as a participatory method and, where possible, children and parents were interviewed separately. Seven children who reported they had received a diagnosis of CRPS‐I and their parents were included (*n* = 14). Transcripts were analyzed using reflexive thematic analysis. Data were analyzed inductively to identify key themes that shaped their experiences before, during, and after diagnosis. Four themes were developed that uncovered a complex journey before, during, and after diagnosis. An overarching theme, Navigating Healthcare, developed alongside three sequential themes: Search for Answers, It's CRPS, and What's Next? This study demonstrates that access to health professionals with the knowledge, experience, and skills to provide rehabilitation for pediatric CRPS may be more important than early diagnosis in influencing CRPS‐I outcomes. Care pathways must include guidance for health professionals on how to explain CRPS‐I so that no family needs to resort to internet searches to explain this complex condition or navigate their child's care.

## 1 Introduction

Complex Regional Pain Syndrome Type I (CRPS‐I) is a chronic pain disorder characterized by severe, disproportionate pain following minor injury without peripheral nerve damage [1]. Rare in children, the largest surveillance study estimates an incidence of 1.14 per 100 000 [2]. CRPS‐I predominantly affects girls in early adolescence, with the mean age of onset being between 11 to 13 years old [2, 3, 4, 5, 6, 7]. CRPS‐I significantly impacts children and parents, disrupting education, social activities, and family life [5]. CRPS‐I can affect both the upper and lower extremities in children; however, it more commonly presents in the lower limb, unlike in adults [4, 8, 9, 10, 11, 12]. In children, the affected limb is typically cold, blue or purple in color, with trophic changes being rare [2, 10, 13]. In contrast, adults more frequently present with a warm, red, swollen limb [9, 10]. Children have been reported to demonstrate a more favorable prognosis and higher functional outcomes than adults [11]. Specifically, only 5.4% of adults were symptom‐free at 12‐month follow‐up [14], whereas 64%–76% of children were reported to be symptom‐free at clinical follow‐up periods ranging from 1 to 16 months [15].

Early diagnosis is crucial to reduce pain‐related disability, psychological distress, and poor outcomes [2, 16]. A current concern is that the diagnostic process for pediatric CRPS‐I remains undefined; clinicians are advised to rely on their expertise, supplemented by diagnostic tools [17]. Investigations, including blood tests, imaging, and electromyography, are used to exclude other conditions rather than confirm CRPS‐I, often leading to multiple referrals and delays in getting a diagnosis [18]. The Budapest criteria are considered the superior diagnostic tool in adults, exhibiting high sensitivity (99%) and respectable specificity (68%) [19]. However, no validated diagnostic tools currently exist for children [20]. When the Budapest Criteria were applied to children, they demonstrated poor sensitivity (64%) but good specificity (89%) [13]. These findings further reinforce concerns that adult‐based diagnostic criteria may be inappropriate for children, supporting the development of pediatric‐specific tools. For example, the Pediatric PainSCAN screening tool is under development to screen for neuropathic pain and CRPS in children [17]. The lack of validated diagnostic tools for pediatric CRPS‐I, coupled with the absence of a diagnostic test, results in delays to a diagnosis.

Limited research to guide clinical pathways [21] and delays to diagnosis make CRPS‐I a condition particularly susceptible to diagnostic uncertainty [22], a recognized issue in pediatric chronic pain literature [23, 24], with Neville et al. [24]. highlighting that long waits fueled distress, mistrust in the medical system, and fear regarding the credibility of a diagnosis. Building upon this, Jordan et al. [23]. noted that parents felt “in limbo” during this waiting period, feeling as though their lives were indefinitely on hold. While these studies highlight the emotional toll of uncertainty, general chronic pain literature involving children with headache, abdominal pain, and widespread pain presentations may not capture specific challenges that exist for children with CRPS‐I. Logan et al. [6]. reported that children with CRPS‐I experienced greater functional impairment than children with back pain (*p* < 0.001). However, school absenteeism was significantly lower in children with CRPS‐I than among those with headache (*p* < 0.05) or abdominal pain (*p* < 0.001) [6]. While early diagnosis is linked to improved outcomes regarding diagnostic uncertainty, the diagnostic experience itself remains unexplored.

This study aimed to bridge this gap by exploring children's and parents' experiences of assessment and diagnosis of CRPS‐I. This aligns with The Lancet's call for patient‐centered pediatric pain research [25], prioritizing children's and parents' experiences and care needs when developing diagnostic pathways.

## 2 Methods

### 2.1 Study Design

This qualitative secondary analysis addressed the research question: *How is the assessment and diagnosis of CRPS‐I experienced by children (11–18 years old) and their parents?* Three objectives were (1): to describe how the diagnosis of CRPS‐I was reached by clinicians (2) to explore the impact of a diagnosis of CRPS‐I, including any perceptions of change pre‐ and post‐diagnosis; and (3) to explore similarities and differences between children's and parents' experiences of receiving a diagnosis of CRPS‐I.

Secondary analysis is an established methodology [26] that efficiently repurposes existing data to address new research questions [27]. Interviews conducted during the primary study incorporated timeline‐based reflections on the onset and early stages of treatment, generating detailed diagnostic narratives. These data were well suited to addressing the aim of the secondary analysis. To mitigate researchers lacking first‐hand knowledge of the data [27], this research was completed with the primary study author, and express consent for secondary analysis was given at the time of data collection. Due to the limited number of children affected by CRPS‐I, secondary analyses enabled the inclusion of this hard‐to‐reach and under‐represented population, whilst minimizing the need to re‐disclose sensitive and potentially distressing information.

The primary study interviewed 21 children [28] treated for chronic musculoskeletal pain and 21 parents [29]. Informed by Q methodology [30, 31], the primary study aimed to explore the outcomes that children and parents considered important during multidisciplinary treatment. Interviews were semi‐structured using a timeline participatory method [32]. The current secondary analysis of interview transcripts and timeline drawings included a subset of these participants (*n* = 14, 7 children and 7 parents) who self‐reported their own or their child's diagnosis of CRPS‐I. Ethical approval for the secondary analysis was granted by the University of Southampton Faculty of Environmental and Life Sciences Ethics Committee (ERGO‐II‐97301).

### 2.2 Participants And Recruitment

The primary study recruited children (11–18 years old) receiving National Health Service (NHS) treatment for chronic musculoskeletal pain, and their parents. To be included in the study, children needed to fulfill the chronic primary pain definition of having pain lasting over three months, accompanied by significant distress or disability that cannot be explained by another diagnosis [33]. Furthermore, they needed to be currently receiving multidisciplinary treatment or be within one month of discharge. Included parents were primary caregivers of a child who fulfilled these criteria. The exclusion criteria were children with long‐term disability and children or parents unable to communicate in English or who had health conditions that prevented them from being able to report their experience. These exclusions were applied due to the absence of resources for translation and because the interview explored participants' perspectives of a future desired treatment endpoint. For children with long‐term disabilities, such an endpoint may not be anticipated or an appropriate expectation. For the secondary analysis, only transcripts and timelines from children and parents who self‐reported receiving a CRPS‐I diagnosis were included. The diagnosis was not verified through medical records or by clinical staff who were unaware of the child's participation in the study.

Recruitment for the primary study occurred in two English hospitals between May 2018 and April 2019. Healthcare professionals acted as gatekeepers by screening and inviting eligible participants. This was initially done through a single mailing, and then, subsequently, children and parents were invited by clinicians during in‐person clinics. To achieve a purposive sample and maximize variation in age, sex, and treatment stage, the last author (RJ) kept the health professionals informed about gaps in inclusivity and attended clinics most likely to fill the gaps, e.g., younger patients or new patient clinics. RJ remained available in a separate room to discuss the study if eligible children and parents chose to. The presence in clinics meant RJ was able to recruit potential participants with particular characteristics, depending on the data collected in real time, with a view to gaining maximum variation sampling as far as possible.

Eligible children and parents contacted the researcher directly by email or telephone if they wanted to take part; they had the opportunity to ask questions and had at least 48 h after receiving the study information before providing written consent. Parents could participate independently of their child, children could participate without parental involvement, and child–parent dyads could also participate. In accordance with Ethics Committee guidance, children provided written consent, with parents informed of their participation, applying Gillick Competence [34] to promote their involvement. Parents provided written consent for their own participation. Recruitment was stopped when the purposive sampling was deemed to have adequate informational power based on sample characteristics and variation across cases, and no new themes were identified [35].

### 2.3 Data Collection

RJ completed all the semi‐structured interviews and was introduced to participants as a researcher. Although a physiotherapist, she did not have any prior relationship with any participant and was not working clinically at the recruiting hospital sites. Interviews incorporated a timeline participatory activity [32, 36] where, through the interview, participants were invited to draw a timeline of their healthcare experience from when the problem first started. This method was introduced to RJ within a session led by Dr. White at a British Psychological Society seminar series [37]. The interview schedule used with children and parents (File S1) was developed in collaboration with two patient representatives and incorporated the timeline drawing instructions provided in the Save the Children Norway toolkit [38]. The timeline drawing offered an element of data triangulation combining verbal and written accounts. Interviews were audio‐recorded and transcribed verbatim, and reflective notes were made immediately following the interview. Following recommendations from patient involvement during the research design, participants chose the interview setting (hospital, home, via telephone or using online methods) at a time that suited them. Participants' choices of interview setting were influenced by the need to fit interviews around normal routines and to select a time and location that allowed experiences to be shared openly without being overheard. Where possible, children and parents were interviewed separately, with children being interviewed first to emphasize the value of their perspectives, as studies indicate children may perceive their opinion as less significant than that of their parents [39]. When requested by the child, a parent was permitted to be present during the interview to support the child's participation. In these instances, parents were asked not to contribute during the child's interview and were subsequently interviewed separately at a different time. The participant information sheet and consent form explained the limits of confidentiality, including the legal obligation to report any disclosures that raised safety concerns. If children or parents raised sensitive topics that caused potential distress without posing a safety risk, they were offered the option to pause or end the interview. Where appropriate, the researcher was equipped to provide contact details for an external support service or suggest the participant speak to their clinical team.

### 2.4 Data Analysis

Throughout data collection and analysis, RJ maintained a reflexive diary and reflected on the potential influence of her professional background on the research process. The reflective diary was discussed with the wider research team in the primary study to promote transparency and trustworthiness in the data analysis.

For the secondary analysis, the transcripts and timeline drawings were analyzed at a semantic level using the six stages of reflexive thematic analysis [40, 41, 42]. This flexible method operates without a fixed theoretical framework [40] and allowed in‐depth exploration of the narratives to identify key themes related to child and parent experience of CRPS‐I diagnosis. Themes were developed by all three authors, positioned as outsiders to the participant group but with physiotherapy backgrounds, bringing shared contextual knowledge to the analysis. This perspective supported a broader biopsychosocial interpretation of participants' accounts of diagnosis, a largely medical process, while necessitating ongoing reflexive critique of how professional positioning shaped the analysis.

An inductive approach was taken where the main author (EE) coded all the transcripts line by line without the use of computer software. The timeline drawings were analyzed alongside the interview transcripts to support interpretation and inform the analytic focus. Any written information recorded on the timelines that was not discussed during the interviews was transcribed verbatim and incorporated into the analysis. There were no drawings (images) that required analysis. Field notes made by RJ were used for reflection but were not used as part of the analysis. Initially, EE coded a single transcript and corresponding timeline and had the opportunity to reflect on her initial codes and discuss the coding process in a 3‐h meeting with their supervisor (RJ). They then went on to code all the transcripts, making reflections during coding, before grouping similar codes and presenting these potential subthemes in a second 3‐h meeting. During this meeting, the subthemes were analyzed manually (using large flip‐chart paper) and re‐organized and refined iteratively until themes were developed that could be described and evidenced with quotes. In Phase 6 (write‐up), themes were revisited and reflectively discussed with LR, and further refined through the peer review process, which facilitated critical challenge. The Consolidated Criteria for Reporting Qualitative Research (COREQ) guidelines have been used to ensure rigor in the analysis and reporting [43].

## 3 Results

Fourteen participants (7 children and 7 parents) described eight different narratives that involved a child being diagnosed with CRPS‐I. Four narratives were told from the child and parent perspective, one narrative only had the parent perspective, two narratives only had the child's perspective, and one narrative had the child and both parents' perspectives. All interviews were completed in a single (individual) session. Two children chose to have their parent in the room during their interview, and the mother and father of the same child took it in turns to talk but did overlap for periods. Six children chose to be interviewed face‐to‐face, either at home (*n* = 3) or in the hospital (*n* = 3), while one child participated via telephone. Children's interviews lasted an average of 50 min (range 29–70 min). Five parents chose to be interviewed face‐to‐face in their homes, and two parents participated by telephone. Parents' interviews lasted an average of 63 min (range 40–82 min). The depth and richness of the data were comparable across interview settings. Table 1 presents the demographic details of children and parents.

**TABLE 1:** Demographic information of children^a^ and parents.

Characteristic	Results
Sex of child, *n* (%)	
Female	7 (87.5%)
Male	1 (12.5%)
Sex of parent, *n* (%)	
Female	6 (86%)
Male	1 (14%)
Age of child in years and months	
Mean (range)	14.3 (12.1–16.9)
Child's initial pain location, *n* (%)	
Lower Limb	6 (75%)
Upper Limb	2 (25%)
Time reported from onset to diagnosis in months	
Mean (range)	5.4 (1.0–19.0)
Pain duration (time since onset) in months	
Mean (range)	14.6 (6.0–33.0)

^a^ The demographics include details of the child who did not participate but whose parent retold their narrative alone; therefore, totals add to 8 for the children.

**FIGURE 1:**
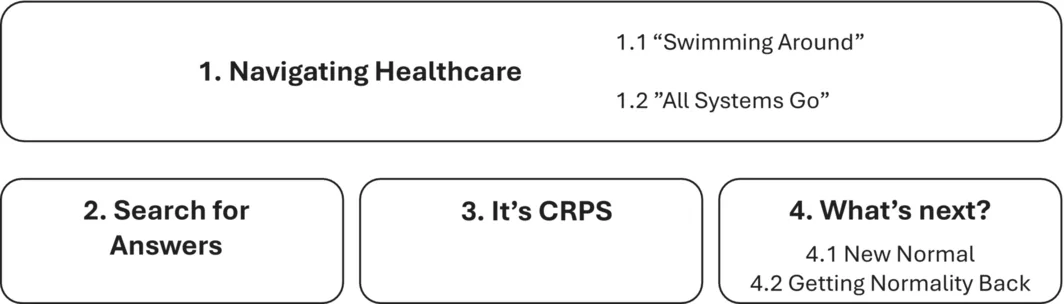
Summary of the 4 themes across the diagnosis journey from the onset of symptoms and assessment to experience post‐diagnosis.

**FIGURE 2:**
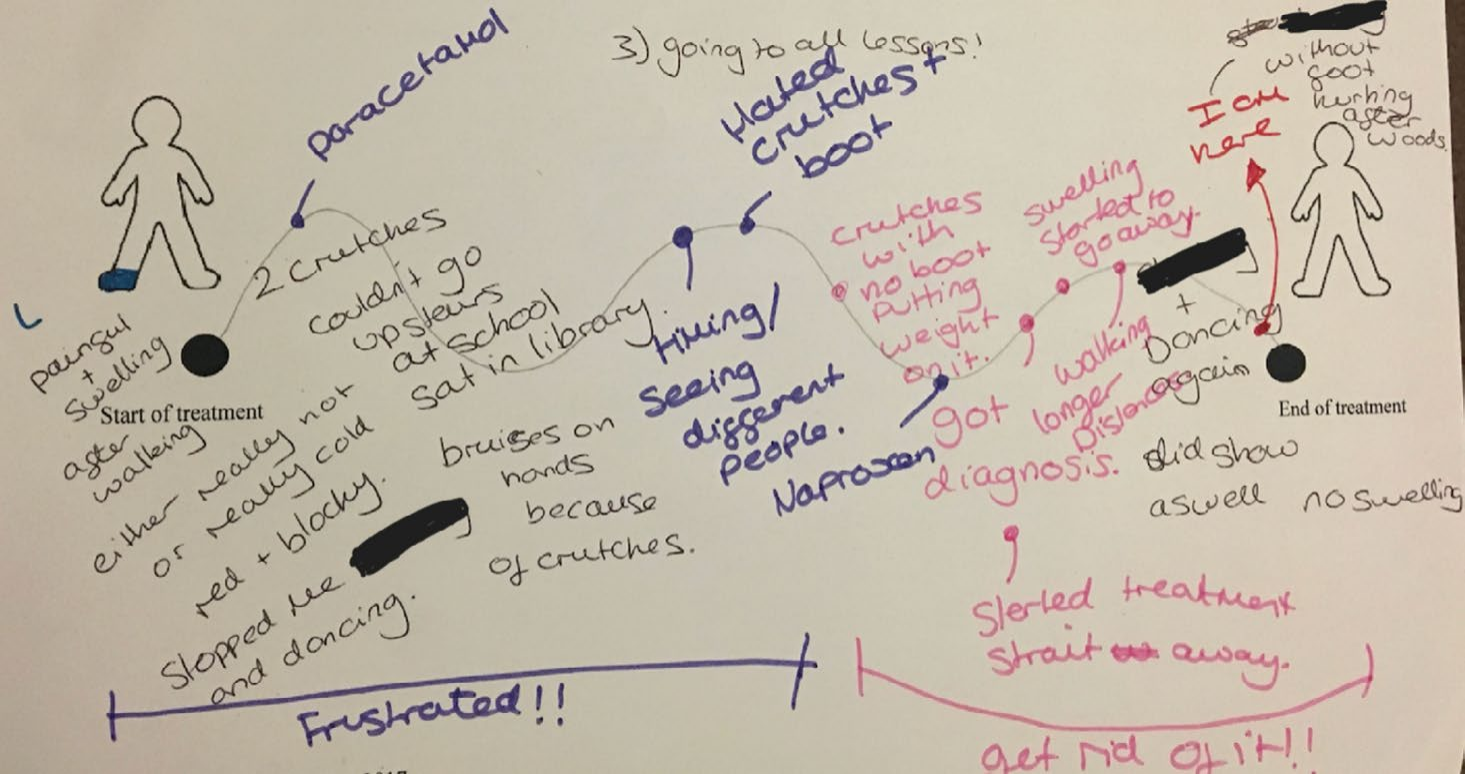
Example of one of the children's timelines. This timeline shows the three distinct phases: Pre‐diagnosis in purple writing (Search for Answers), the point where they were given a diagnosis labeled ‘got diagnosis’ (It's CRPS), and post‐diagnosis in pink writing (What's next?). Aspects of the theme ‘Navigating Healthcare’ can be seen pre‐ and post‐diagnosis, shown by the writing ‘seeing different people [doctors]’ and ‘started treatment strait [sic] away’. The time each person perceived to spend in each of the three phases differed.

From the thematic analysis, four themes were developed, as shown in Figure 1. An overarching theme, Navigating Healthcare, developed alongside three sequential themes: Search for Answers, It's CRPS and What's Next? These themes were developed at different stages of the diagnosis journey and are explored in the order they are presented; this is shown by the timeline example in Figure 2. Pseudonyms for each of the 8 narratives were used.

### 3.1 Theme 1: Navigating Healthcare

This theme demonstrates the crucial role of the healthcare system: How children and parents navigated and accessed services, and the impact of their interactions with health professionals. Navigating healthcare influenced all other themes, shaping experiences before, during, and after diagnosis. Two distinct trajectories developed: “Swimming Around” and “All Systems Go”, though movement between trajectories was observed among some participants.

#### 3.1.1 “Swimming Around”

This subtheme was named from a mother's quote and captures the challenges faced by all families who navigated fragmented and disjointed healthcare either before, after, or both before and after diagnosis.

Charlotte's mother: “…this whole process so far I think I was sort of **swimming around** trying to work out what was going on.”

It describes the experiences of children and parents moving through the health system with no direction or purpose, uncertain whether any action had been taken, and/or stuck in a cycle of being referred between services.

Zoe's Mother: “I would come home and say ‘how was your appointment?’ and they'd go [Zoe and father] there is no change, there is nothing different, I'm being referred to this, or this is going to happen in two weeks…that was quite frustrating”.

Parents seemed to attribute this lack of continuity of care and negative healthcare experiences to healthcare professionals' poor awareness of CRPS‐I and the condition's rarity.

Emily (child): “I'd seen many consultants and physios who had never seen it [CRPS‐I] before and didn't know what to do. They were kind of just guessing and wanted me as something for research to kind of get to know the condition more but…I just wanted to get better; I didn't want to be someone's first case of the condition.”

Although children reported being diagnosed within their local hospital, a group of families reported voids in local service provision and being unable to access trusted information or follow‐up care. Ultimately, parents and children stuck in this ‘swimming around’ phase developed a mistrust in the health system and a sense of hopelessness. To find a way out, parents took the lead, doing their own research, suggesting investigations, services or treatments, and seeking private care to make progress.

#### 3.1.2 “All Systems Go”

This subtheme describes positive healthcare experiences defined by healthcare professional consistency, clear information and action. This led to the provision of a CRPS‐I diagnosis, and subsequent engagement of children and parents in the management plan.

Zoe's mother: “Quite dramatic, changing from orthopedics to rheumatology. It was then like **all systems go**, and everything got put into place…once she knew there was a diagnosis and once she knew what she could and couldn't do…[Zoe's] mindset really changed”

None of the children or parents described experiencing an ideal pathway of care; instead, positive experiences were often described as chance encounters. Families described feeling lucky to have the same professionals who understood the child, could make the diagnosis, and/or reassure them that progress was being made, and the condition was reversible. There appeared to be an overwhelming need from children and parents to find professionals and services who successfully diagnose and treat children affected by CRPS‐I.

Emily's mother: “The relief of speaking to somebody who knew what they were talking about was just, you know, I was like ‘You're the first person that actually understands what's happening.’”

If parents found a health professional or team they trusted, they seemed to become a source of support, and they gained a sense of hope.

Noah's mother: “The support was from the pain clinic…I think the reassurance actually, and also I think telling us that actually you are doing a good job…you feel rubbish, you know…I think really just the support and knowing that he will get out of it”.

Continuity of health professionals appeared to influence children's outcomes pre‐ and post‐diagnosis, with one child who reported waiting 19‐months for a CRPS‐I diagnosis, significantly improving with rehabilitation from a consistent local physiotherapist prior to their diagnosis.

### 3.2 Theme 2: Search for Answers

This theme captured how children and parents perceived the process by which doctors gathered and interpreted medical evidence to arrive at a diagnosis of CRPS‐I. Experiences varied considerably with time until diagnosis, reported to range from 1 to 19 months; longer wait times were linked to stress and slow progress, while shorter wait times to diagnosis were described more positively.

This phase was described as a series of tests and appointments that were experienced as procedural, system‐driven, and clinician‐led. Concern and a search for a diagnosis appeared to dominate parents' narratives, with the need to find the cause of their child's pain. In some cases, there seemed to be a role reversal between parents and medical professionals, where parents researched symptoms and suggested further investigations.

Ada's mother: “I was quite insistent that the MRI was done, then I googled, looked at the Government NHS website, and they said before you diagnose it [CRPS‐I] you also need to do like a muscle test, an EMG, I think it was called. So, I tried to get that done. So, trying to get it diagnosed properly was important to me and seemed less important to the professionals… I think if I hadn't googled, I wouldn't have got all the tests she needed”.

In contrast, children tended to describe this stage with less detail and expressed comparatively less concern.

Ada (Child): “I didn't really know what it was…it was just weird …I was never that worried. I assumed it would go. At the start I didn't think it would last as long as it did, but mum was quite worried.”

The assessment process appeared to depend on how symptoms were presented. Most children reported a traumatic onset of pain, which meant the initial assessment was completed within accident and emergency and/or orthopedics, with only one child being referred to rheumatology for further investigations. Using X‐ray, a fracture was confirmed in one case, and a soft tissue injury was confirmed in another. However, a group of children was initially told they had a fracture and then, in a future appointment, told no fracture had existed, or there was uncertainty as to whether there was an initial fracture. The overriding feature of children who had a traumatic onset was extreme pain and hyperalgesia.

Ella's mother: “[the doctor] took the shoe off and touched it all, and she was properly screaming and crying and saying it really hurts…[the doctor] spoke to his colleague who was apparently his boss…and said ‘actually we've had a look at the x‐ray again and there was never a fracture there’”.

A couple of the narratives described children experiencing spontaneous foot swelling, color change, and temperature change in the presence of extreme pain and hyperalgesia but with no known triggering event. This presentation appeared to lead to more tests and medical opinions in this assessment phase, including from general pediatricians, orthopedic surgeons, pediatric rheumatologists, and vascular surgeons.

### 3.3 Theme 3: It's CRPS

This theme captures the importance of how the diagnosis was delivered by health professionals. There appeared to be two distinct components of the diagnosis delivery: (1) giving the diagnosis a name (CRPS‐I) and (2) having an explanation of CRPS‐I that made sense. While all the narratives described a doctor naming CRPS‐I either in person or in a letter, only two children and their parents reported they received an explanation at the time of diagnosis. In both these cases, a rheumatologist delivered the diagnosis with an explanation, and the experience was described positively, with immediate impact reported by the child.

Emelia (Child): “I finally found out what it was, so that was a good thing…when it goes on for so long you start wondering ‘is it all in my head or something’…It is, but it isn't. There is something there; it's not me making it all up, and so I was really happy”.

Conversely, most narratives described the diagnosis delivery without an explanation.

Charlotte's mother: “We got an appointment back with the orthopedic surgeon…there was a bit of like ‘oh, oh, ok, erm’ and then they just took one look at her leg and said (large sigh) CRPS get hydro sorted asap”.

Lack of confidence in health professionals' knowledge and experience seemed to fuel fear, as did searching CRPS‐I on the internet for both parents and children.

Emily (Child): “The doctor who diagnosed me wasn't very useful. He didn't really know what CRPS was, so he actually told us to go and research it for ourselves… Google can give some pretty extreme cases, which was quite scary”.

In addition, three narratives of the child and/or parent described the experience of being assessed for and receiving the diagnosis as traumatic. They reported feeling their pain was dismissed during assessments, exacerbating the child's distress, pain, and disability.

Chloe (child): “[the doctor] was like pulling my foot around. It was painful, he was like pulling it around and bending my legs…he was a new doctor…that's the day I asked for crutches”.

### 3.4 Theme 4: What's Next

This theme focused specifically on children's and parents' experiences of accessing and engaging with CRPS‐I management following a diagnosis. Participants described varying levels of timely access to services and health professionals able to provide the expected treatment, and these experiences appeared to shape how they adapted to life with CRPS‐I. These differing experiences led to the subthemes ‘new normal’ and ‘getting normality back’.

#### 3.4.1 New Normal

When the diagnosis failed to lead to the required steps for recovery, both children and parents felt trapped in their current circumstances‐ a difficult ‘new normal’. The narratives of children who reported poorer outcomes reported difficulties in accessing care, treatment and services after a diagnosis of CRPS‐I. Even when care was accessed, parents often perceived it as inappropriate for managing CRPS‐I, especially physical rehabilitation. Parents described frustration when the diagnosis failed to lead to clear recovery steps; this appeared to diminish the perceived value of the diagnosis, making parents feel helpless.

Emily's mother: “All the research that I did at that point it did keep coming back to that physio; early physio was one of the main things you needed…we had an appointment at [Hospital 1 and 2]…but the physios had no experience of CRPS, felt like it made it worse. By New Year, the pain was up to her knee.”

For children, prolonged waiting and limited progress appeared to intensify their pain. Both parents and children described a pervasive fear of the pain spreading or worsening, creating a cycle of catastrophizing. For a small group of children, repeated failures to improve their condition seemed to diminish hope, and a multidisciplinary pain service was seen as the only option.

Charlotte (child): “When we got a letter back from the pain service saying they would accept me as a patient…that was quite a good feeling, like ‘wow, we've actually managed to get some help’”.

#### 3.4.2 Getting Normality Back

This subtheme highlighted the transformative impact of early and structured interventions following a CRPS‐I diagnosis. The narratives of the four children who reported a complete resolution of CRPS‐I symptoms and/or significant functional improvement all described early, local access to physical rehabilitation, with one narrative also reporting regular psychology support. Young people described relationships with these clinicians as consistent, positive, and trusting.

Many on this path described the diagnosis as a turning point; children and parents reported positive outcomes and a return of normality. Those who experienced delays before diagnosis reported better outcomes if they transitioned onto this positive trajectory. Timely access to health professionals knowledgeable about CRPS‐I, who built on the child and parent's understanding of the diagnosis and provided reassurance that they were on the right path, could foster a belief in recovery.

Zoe (child): “In my first physio session…[the physiotherapist] told me everything about [CRPS], they explained it really clearly, and I just relaxed more… which helped me get through it quicker”.

For a group of children, access to child and mental health services post‐diagnosis was important for engagement in rehabilitation and ongoing management.

Charlotte (child): “It was now more ‘we need to deal with your emotions rather than your physio’ because then physio will become a lot easier”.

## 4 Discussion

This study explored the experiences of children and parents when receiving a diagnosis of pediatric CRPS‐I, uncovering a complex journey before, during, and after diagnosis. Narratives highlighted key factors shaping this experience, including healthcare continuity, diagnostic uncertainty, communication at diagnosis, and access to treatment. A qualitative study by Griffiths et al. [44]. exploring diagnosis experiences of adults experiencing CRPS‐I found similar findings, suggesting a life course approach to diagnosis and management may be beneficial. While prior pediatric research highlights the importance of early diagnosis as crucial for better outcomes [2, 3], recent work by Mesaroli et al. [16]. highlights the complementary role of early intervention. Building upon this, the present study suggests post‐diagnosis care appears equally, if not more, crucial than early diagnosis. Despite a challenging start, marked by prolonged waiting in ‘search for answers’ or poor communication at the point of diagnosis, positive outcomes appeared to be still possible when the diagnosis led to a timely referral to rehabilitation. Therapists (including physiotherapists and occupational therapists), perceived to have the skills and knowledge of pediatric CRPS‐I, were able to explain the condition and provide appropriate treatment, often in conjunction with child and adolescent mental health support where needed. These findings support conceptualizations of diagnosis as a relational process rather than a discrete clinical act, aligning with sociological theories that frame diagnosis as socially situated and shaped through interactions over time [45, 46]. From this perspective, diagnosis does not merely name a condition but contributes to how individuals construct their understanding of illness and what they perceive to be appropriate and meaningful forms of treatment [45].

A key challenge experienced by all narratives was navigating a fragmented healthcare system. This process left families feeling abandoned and exhausted, reinforcing Carter's [47] finding of “referral fatigue”. Despite being over 20 years since this concept was published, it remains a persistent problem and describes a cycle of repeated referrals that raise, then shatter hopes for diagnosis and treatment, an experience mirrored in the narratives. This is supported by pediatric chronic pain literature, which describes long waiting periods and multiple unsuccessful referrals as significant stressors for children and their parents [23, 24, 47, 48, 49]. However, the current study highlights the importance of “referral fatigue” not only as a pre‐diagnosis struggle but also post‐diagnosis. This supports findings from a meta‐ethnography that found that finding the cause of chronic pain and pursuing effective management led to repeated visits to health services [49]. These findings highlight the impact of fragmented care and evidence the crucial role of continuity before a diagnosis, and beyond it, to drive better outcomes.

The average time between the onset of symptoms and diagnosis was 5.4 months, but some children experienced delayed diagnosis, which is similar to findings from a Canadian surveillance study of CRPS [2]. The current study indicates that diagnostic uncertainty, where there is a ‘subjective perception of an inability to provide an accurate explanation of a patient's health problem’ ([50] p103), is a key feature of diagnosing CRPS‐I. Notably, in the current study, parents and children had differing priorities during this pre‐diagnosis period. Parents prioritized securing a diagnosis for their child's pain, a pattern seen in chronic pain literature [23, 24]. The initial uncertainty surrounding the presence of a bone fracture appeared to be a key finding and a potential early indicator of developing CRPS‐I that warrants further exploration. In contrast to chronic pain literature, the current study suggested that once the diagnosis of CRPS‐I had been given, parents did not continue searching for an alternative diagnosis. Similar to experiences of clinicians [51], additional tests with normal results were reassuring to parents, as they confirmed the correct diagnosis had been reached; however, mixed messages regarding the results of investigations perpetuated uncertainty. The current study emphasizes the need to involve and explain the diagnostic process for CRPS‐I to children and their parents, continue to develop diagnostic tools to reduce reliance on investigations [17], and ensure clinicians are adequately trained to provide a CRPS‐I diagnosis. Transparent discussions could foster trust and engagement and help manage parent expectations, so they feel supported in the process.

How the diagnosis of CRPS‐I was delivered and received by children and parents mattered. Most narratives described hesitant or unclear communication at the point of diagnosis. Only a few accounts reported that the diagnosis was given with a clear explanation. This highlights the potential need for clinicians to receive training in how to explain CRPS‐I to avoid families having to independently research the condition on the internet. Future research could develop a credible explanation with children and parents, a process that has been completed for pediatric knee pain [52]. While broader diagnoses such as ‘chronic pain’ or ‘medically unexplained symptoms’ have been linked to feelings of invalidation and mistrust [23, 24, 53], the current study suggests a CRPS‐I diagnosis may contribute to a greater sense of legitimacy. Interestingly, this study found that uncertainty stemmed more from poor communication and lack of an explanation than the diagnosis itself. This could be rectified if a health professional who had experience of pediatric CRPS‐I could explain the diagnosis in a way that made sense and give children and parents hope of recovery and realistic timescales.

Time‐to‐treatment appeared to play a significant, if not greater, role than time‐to‐diagnosis in influencing outcomes. Delays in care were reported to result in worsening symptoms and distress, yet swift, appropriate interventions appeared to mitigate earlier delays or poor diagnosis delivery. This supports the International Association for the Study of Pain (IASP) task‐force on waiting times that recommends a 1‐week waiting time for children in the acute phase of CRPS‐I to access appropriate care [54]. While research on time‐to‐treatment in pediatric CRPS‐I is limited, a systematic review by Bean et al. [55]. found that a delay in receiving treatment was associated with poor outcomes in adults. The current study suggests prompt intervention may influence outcomes, even after a prolonged and difficult start to attain a diagnosis. The role of trusted health professionals providing rehabilitation, pre‐ and post‐diagnosis, appeared to be an important, but under‐researched area of enquiry. Overall, it was apparent that healthcare systems need structured care pathways that provide immediate access to services that have expertise in treating children affected by CRPS‐I. Readily available referral pathways will equip professionals with the knowledge of where to refer patients, even if pediatric CRPS‐I is unfamiliar to them. Continuity of care from the initial point of contact is essential in adopting a child‐ and family‐centered approach, necessary to accommodate the apparent differing needs between parents and children.

Limitations of this study should be noted. Participants were recruited from two publicly funded English hospitals; experiences of children and parents from different countries or privately funded services may have differed. Secondary analysis potentially limited the coverage of the new research question [26] meaning broader diagnostic perspectives may not have been fully captured. The sample had a predominance of girls and mothers over boys and fathers. While this aligns with the known presentation of CRPS‐I and existing samples in chronic pain research, it may limit insight into boys' and fathers' experiences. Not collecting data on participants' socioeconomic status and ethnicity limits the ability to fully understand the diversity within the sample and will be included in future research. Additionally, thematic analysis was conducted at a semantic level, focusing more on explicit content than deeper emotional or unspoken diagnostic concerns, and this is worthy of further research. Finally, this study focused on children with CRPS‐I and their parents, excluding siblings and healthcare professionals despite indications in the narratives that they were also impacted. Future research should explore these perspectives to provide a broader understanding of the impact of a CRPS‐I diagnosis and its management.

In conclusion, the current study highlights how continuity of care, diagnostic uncertainty, communication, and access to treatment all shape the impact of a pediatric CRPS‐I diagnosis. Early diagnosis and timely access to health professionals with the required knowledge and experience of pediatric CRPS‐I is crucial. No family should have to use Google to navigate their way through the health system to receive an explanation and support for their child's pain. There is important learning to be taken from services that are perceived to positively deliver the diagnosis and provide access to experienced health professionals. Understanding this diagnostic process is important to include in the development of best practice guidance and healthcare pathways to improve outcomes for children experiencing CRPS‐I.

## Funding

The authors have nothing to report.

## Ethics Statement

University of Southampton Ethics approval was obtained for the secondary analysis (Ref: ERGO‐II‐97301), and the National Health Service (NHS, Leeds, UK) Research Ethics Committee (Ref: 18/SC/0138) approved the primary program of research.

## Consent

All participants have given written consent to participate.

## Conflicts of Interest

The authors declare no conflicts of interest.

## Supporting information


**File S1:** Semi‐structured interview schedule incorporating a timeline participatory method.

## Data Availability

Research data are not shared.
