# Supplementary material for: “Google It”: Experiences of Children and Parents During the Assessment and Diagnosis of Complex Regional Pain Syndrome Type I
Source: Paediatr Neonatal Pain. 2026 Sep 5;8(3):e70045. doi: 10.1002/pne2.70045 (PMC13545539; doi:10.1002/pne2.70045)
Supplement: Supplementary file 1 [file PNE2-8-e70045-s001.docx]

**Supplementary file 1- Semi-structured interview schedule incorporating a timeline participatory method.**

This interview schedule was used across different interview medium (face-to-face, online or telephone).

**For children**

| **Broad Question** | **Probing Follow-Up Questions** |
| --- | --- |
| **Looking at the empty timeline can you first go to the “START” point.**  1. Can you tell me about yourself at the start of your treatment when you first came to hospital for pain treatment? | What was your pain like? Where did it affect?  What were you able to do?  How did you spend your time?  What were you saying?  How did you feel? |
| **Now go to the “END’ point**  2. At the END, describe yourself at the end of an IDEAL treatment when you no longer need to come to hospital. (this may be in the future). | What would you be doing?  Where would you be spending time?  What would you be saying?  How would you be feeling?  What do you think your parents and family would say? |
| **Joining the dots**  3. Now consider your journey from the start of treatment until the end and join the “Start” and “End” dots to show what your journey has looked like so far and what it might look like into the future.  4. Now mark the line with the point where you are now on this journey and write “I AM HERE” and an arrow. | Add any important events that have happened or are happening in your life. |
| **Positive Signs**  5. How would you know if a treatment is working? Write positive signs on your treatment timeline. | What are the early signs/ final signs a treatment is working?  What changes make the biggest difference?  Do you think health professionals/ parents notice these changes?  Tell me more about x that you mentioned?  Can you tell me more about why you chose these. |
| **Negative signs**  6. How would you know a treatment was not working? Draw negative signs on your treatment timeline. | What are the early/late signs that tell you treatment is not working?  What changes cause the most impact and would stop you continuing the treatment?  Do you think health professionals/ parents notice these changes?  Tell me more about x that you mentioned?  Can you tell me more about why you chose these. |
| **Change over time**  7. If we spoke at the start of treatment would you have said anything different? |  |

For parents

| **Broad Question** | **Probing Follow-Up Questions** |
| --- | --- |
| **Looking at the empty timeline can you first go to the “START” point.**  1. Can you tell me about your child at the start of their treatment when you first came to hospital for their pain? | What was their pain like? Where did it affect?  What were they able to do?  How did they spend their time?  What were they saying?  How did they feel?  Were you and other family members affected? |
| **Now go to the “END’ point**  2. At the END, describe your child at the end of an IDEAL treatment when they no longer need to come to hospital. (this may be in the future). | What would they be doing?  Where would they be spending time?  What would they be saying?  How would they be feeling?  Would you and other family members be affected? |
| **Joining the dots**  3. Now consider your child’s journey from the start of treatment until the end and join the “Start” and “End” dots to show what your journey has looked like so far and what it might look like into the future.  4. Now mark the line with the point where you are now on this journey and write “I AM HERE” and an arrow. | Add any important events that have happened or are happening in your child’s life. |
| **Positive Signs**  5. How would you know if a treatment is working for your child? Write positive signs on your treatment timeline. | What are the early signs/ final signs a treatment is working?  What changes make the biggest difference?  Do you think health professionals/ children notice these changes?  Tell me more about x that you mentioned?  Can you tell me more about why you chose these. |
| **Negative signs**  6. How would you know a treatment was not working for your child? Draw negative signs on your treatment timeline. | What are the early/late signs that tell you treatment is not working?  What changes cause the most impact and would stop you continuing the treatment?  Do you think health professionals/ children notice these changes?  Tell me more about x that you mentioned?  Can you tell me more about why you chose these. |
| **Change over time**  7. If we spoke at the start of treatment would you have said anything different? |  |
